# Retraction Note: Verbena officinalis (VO) leaf extract as an anti-corrosion inhibitor for carbon steel in acidic environment

**DOI:** 10.1038/s41598-026-45155-3

**Published:** 2026-03-23

**Authors:** Abd El-Aziz S. Fouda, Ahmed F. Molouk, Mohamed F. Atia, Ahmed El-Hossiany, Mohamed S. Almahdy

**Affiliations:** 1 Department of Chemistry, Faculty of Science, Mansoura University, Mansoura, 35516 Egypt; 2 Department of Chemistry, Faculty of Science, Tanta University, Tanta, Egypt; 3 Delta for Fertilizers and Chemical Industries, Talkha, Egypt

Retraction of: *Scientific Reports* 10.1038/s41598-024-65266-z, published online 12 July 2024

The Editors have retracted this Article.

After publication, concerns were raised that the data shown in Figure 12 was previously published in a number of papers with some shared authorship, where the data was described differently e.g.^1–6^. Given the nature and the extent of this issue the Editors no longer have confidence in the integrity of the data reported in this Article.

None of the Authors responded to the Editors correspondence about this retraction.
